# Infection Prevalence and Antibiotic Resistance Levels in *Ureaplasma urealyticum* and *Mycoplasma hominis* in Gynecological Outpatients of a Tertiary Hospital in China from 2015 to 2018

**DOI:** 10.1155/2021/8842267

**Published:** 2021-01-13

**Authors:** Wei Zhang, Lijuan Li, Xuelian Zhang, Hongshu Fang, Huajian Chen, Changxian Rong

**Affiliations:** ^1^Department of Clinical Laboratory, The Fourth People's Hospital of Chongqing, Chongqing 400014, China; ^2^Department of Gynecology, The Fourth People's Hospital of Chongqing, Chongqing 400014, China

## Abstract

The aim of this study was to estimate the *Ureaplasma urealyticum* and *Mycoplasma hominis* infection prevalence and antibiotic resistance levels in gynecological outpatients. Clinical characteristics and laboratory data of gynecological outpatients of the Fourth People's Hospital of Chongqing from 2015 to 2018 were retrospectively analyzed. Antibiotic resistance levels in *U. urealyticum* and *M. hominis* were defined by a commercial *Mycoplasma* kit for antibiotic susceptibility testing. Univariate analysis and multivariate logistic regression analysis were performed to evaluate risk factors associated with *Mycoplasma* isolation. Comparisons of yearly distributions and resistance rates were assessed by chi-square tests. Fifty-six percent of gynecological outpatients were positive for *U. urealyticum*, and 11.02% were positive for *M. hominis*. In the univariate analysis, women aged 30–39 years or with a history of pregnancy or gynecological diseases had an increased risk for *Mycoplasma* isolation, while women who were postmenopausal or had an education level of undergraduate degree or above had a decreased risk of *Mycoplasma* isolation. In the multivariate logistic regression model, an independent risk factor for *Mycoplasma* isolation was a history of gynecological diseases, while a bachelor's degree, master's degree, or above were protective factors against *Mycoplasma* isolation. There were distinctly gradual increases in the positivity rates of *U. urealyticum* and *M. hominis* from 2015 to 2018 and an overall increasing trend of resistance to ten antibiotics among *U. urealyticum* and *M. hominis*. The top three antibiotics associated with resistance were ofloxacin, sparfloxacin, and levofloxacin. Doxycycline, josamycin, and minocycline were preferred because they had the lowest levels of resistance. Increases in the prevalence of infection and antibiotic resistance in *U. urealyticum* and *M. hominis* were observed from 2015 to 2018, clearly confirming the necessity to monitor the standardized administration of antibiotics.

## 1. Introduction

*Ureaplasma urealyticum* and *Mycoplasma hominis* belong to the class Mollicutes, genus *Mycoplasma*, which are the smallest known free-living microorganisms [1]. Genital *Mycoplasma* mainly consisting of *U. urealyticum* and *M. hominis* is found among the cervical flora of approximately 70% of women of child-bearing age [2]. Asymptomatic infections by *U. urealyticum* and *M. hominis* have been proven to be associated with increased risks of urogenital tract inflammation [3], female infertility [4], and adverse pregnancy outcomes [5]. Unlike conventional bacteria, *Mycoplasma* lacks a cell wall and thus is resistant to antibiotics that interfere with cell wall synthesis, such as penicillins, cephalosporins, and vancomycin, but they are usually susceptible to antibiotics that inhibit protein synthesis or suppress topoisomerases, such as quinolones, tetracyclines, and macrolides [6, 7]. Some species of *Mycoplasma* are innately or selectively resistant to an antibiotic, while others are not. Some species initially sensitive to an antibiotic can become resistant. The extensive abuse of quinolones has led to a gradual increase in resistance of *U. urealyticum* and the incidence of fluoroquinolone-resistant strains in many countries [8–10]. Furthermore, the high detection rate and poor therapeutic efficacy for *U. urealyticum* and *M. hominis* are great challenges for clinicians and cause heavy economic and mental burdens on patients [11]. Here, we discuss the evolution of antibiotic resistance in *U. urealyticum* and *M. hominis* in gynecological outpatients from 2015 to 2018.

## 2. Methods

This retrospective study was performed at the Fourth People's Hospital of Chongqing (Chongqing, China). Ethical approval was obtained by the Ethics Committee of the Fourth People's Hospital of Chongqing. Routine gynecological examinations were conducted in accordance with regulations and guidelines. The clinical characteristics of outpatients in the Department of Gynecology from January 1, 2015, to December 31, 2018, including age, marital status, education level, occupation, smoking status, drinking status, gravidity, parity, menopause status, and history of gynecological diseases, were collected from the Hospital Information System (HIS).

Endocervical specimens were examined by the Department of Clinical Laboratory. All specimens were processed within one hour after collection. The identification and antibiotic susceptibility of *U. urealyticum* and *M. hominis* were determined by a commercial *Mycoplasma* ICS kit (Lizhu Biotech Co., Ltd., Zhuhai, China) according to the manufacturer's guidelines and standard operating procedures. All inoculated strips were incubated at 37°C in a CO_2_ incubator and then observed for color changes. The results of *U. urealyticum* and *M. hominis* detection were interpreted after 48 hours of incubation. The strips provided information on susceptibility to ten antibiotics including tetracycline, ofloxacin, doxycycline, josamycin, sparfloxacin, roxithromycin, minocycline, levofloxacin, clarithromycin, and azithromycin, at two concentrations, with three interpretations: susceptible (*S*), intermediate (*I*), and resistant (*R*). The breakpoints were as follows (mg/L): tetracycline *S* ≤ 4, *R* ≥ 8; ofloxacin *S* ≤ 1, *R* ≥ 4; doxycycline *S* ≤ 4, *R* ≥ 8; josamycin *S* ≤ 2, *R* ≥ 8; sparfloxacin *S* ≤ 1, *R* ≥ 4; roxithromycin *S* ≤ 1, *R* ≥ 4; minocycline *S* ≤ 4, *R* ≥ 8; levofloxacin *S* ≤ 1, *R* ≥ 4; clarithromycin *S* ≤ 1, *R* ≥ 4; azithromycin *S* ≤ 1, *R* ≥ 4.

Statistical analysis was performed using SPSS software 22.0. Prevalence and antibiotic resistance rates were calculated as frequencies (*n*) and percentages (%). Associations between clinical characteristics and *Mycoplasma* isolation were measured by the chi-square test, as well as associations between year and *Mycoplasma* isolation and associations between year and antibiotic resistance. Multivariate logistic regression analysis was used to assess the risk factors associated with *Mycoplasma* isolation. A *P* value <0.05 was considered to be statistically significant.

## 3. Results

### 3.1. Clinical Characteristics of Gynecological Outpatients

A total of 5670 gynecological outpatients from 2015 to 2018 were included in this study, and the overall prevalence of *Mycoplasma* isolation was 56.53%. The univariate analysis results of clinical characteristic associations are shown in Table 1. Approximately, half of the gynecological outpatients with *Mycoplasma* isolation were in the 20–29 year age range; however, *Mycoplasma* isolation had the highest frequency in the 30–39 year age range. Gynecological outpatients who were postmenopausal or had an undergraduate degree or above were less likely to develop *Mycoplasma* infection than their counterparts. The risk for *Mycoplasma* isolation was significantly increased in patients with a history of pregnancy or gynecological diseases. No association was found between *Mycoplasma* isolation and marital status, occupation, smoking status, drinking status, or parity.

### 3.2. Risk Factors for *Mycoplasma* Isolation

The risk factors for *Mycoplasma* isolation based on the multivariate logistic regression analysis are shown in Table 2. In the multivariate model, an independent risk factor for *Mycoplasma* isolation was a history of gynecological diseases, while education levels of bachelor's degree, master's degree, and above were protective factors against *Mycoplasma* isolation. In addition, there were no associations between *Mycoplasma* isolation and age, gravidity, or menopausal status.

### 3.3. Yearly Distributions of *U. urealyticum* and *M. hominis* from 2015 to 2018

The distributions of *Mycoplasma* isolation between 2015 and 2018 are shown in Table 3. Among 5670 gynecological outpatients, 3205 (56.53%) were positive for *U. urealyticum*, and 625 (11.02%) were positive for *M. hominis*. There was a distinctly gradual increase in the positivity rates for *U. urealyticum* and *M. hominis* from 2015 to 2018. Furthermore, the prevalence rates of both *U. urealyticum* and *M. hominis* had obvious differences (*P*=0.018 and *P*=0.003, respectively) according to the yearly distributions.

### 3.4. Antibiotic Resistance of *U. urealyticum* and *M. hominis* from 2015 to 2018

Table 4 shows the progression of antibiotic resistance of *Mycoplasma* from 2015 to 2018. There was an overall increasing trend of resistance to ten antibiotics among *U. urealyticum* and *M. hominis*; the resistance rates to ofloxacin as well as sparfloxacin, roxithromycin, levofloxacin, clarithromycin, and azithromycin were significantly different among the four years. The top three antibiotics associated with resistance were ofloxacin, sparfloxacin, and levofloxacin, with total resistance rates of 61.84%, 51.54%, and 40.50%, respectively. However, the resistance rates to tetracyclines, including tetracycline, doxycycline, and minocycline were always below 3%, while those to macrolides, including roxithromycin, clarithromycin, and azithromycin, were approximately 20%, except for josamycin, which had a less than 2% resistance rate. Therefore, doxycycline, josamycin, and minocycline are recommended because they had relatively low resistance rates.

## 4. Discussion

This study was performed in a tertiary hospital in Chongqing, China, to evaluate the infection prevalence and antibiotic resistance levels of *Mycoplasma* in gynecological outpatients, as well as the clinical characteristics and risk factors. Overall, in the present study of 5670 gynecological outpatients, we found that 56.53% of outpatients were positive for *Mycoplasma*. Education level and history of gynecological diseases were independently associated with *Mycoplasma* isolation, and a history of gynecological diseases was a risk factor for *Mycoplasma* isolation, while a bachelor's degree, master's degree, and above were protective factors against *Mycoplasma* isolation. Although approximately half of the gynecological outpatients with *Mycoplasma* isolation were 20–29 years old, *Mycoplasma* isolation occurred most frequently in the 30–39-year age group, consistent with one study that showed that the proportions of *U. urealyticum* and *M. hominis* detected in the 30–39-year age group were higher than those detected in the other age groups [12]. A prospective, cross-sectional study found that *Mycoplasma* infection had statistically significant correlations with more than 4 sexual partners, coinfection with other sexually transmitted pathogens, inconsistent contraceptive use, abortion, and history of gynecological diseases [13]. However, no associations between *Mycoplasma* infection and marital status, occupation, smoking status, drinking status, or parity were found in this study.

The detection rate significantly increased by year for *U. urealyticum*, from 53.80% in 2015 to 59.33% in 2018, and for *M. hominis*, from 8.71% in 2015 to 12.42% in 2018. Several studies with similar aims have been performed in China, although they were limited to various years and specific populations. In a tertiary hospital in Beijing, China, between 2009 and 2013, the prevalence rates of *U. urealyticum* and *M. hominis* in female outpatients were 33.1% and 2.6%, respectively [12]. A prospective study in Baoding, China, from 2013 to 2014 found a *U. urealyticum* prevalence of 57.60% in female outpatients with urogenital infections [14]. A community-based cross-sectional study in Shanghai, China, from 2009 to 2014 showed that the *U. urealyticum* infection rate in vaginal secretions was 43.62% in women with urinary tract infections [15]. The wide-ranging prevalence of *U. urealyticum* and *M. hominis* in previous studies could be attributed to variations in study designs, study populations, sampling sites, and laboratory methods [16].

Quinolones are extensively used for the treatment of urogenital and gastrointestinal infections; they prevent pathogen DNA from unwinding and duplicating by inhibiting the type II topoisomerase DNA gyrase and topoisomerase IV [17]. A large number of studies have shown that the *Mycoplasma* resistance rate to quinolones is much higher than those to other antibacterials, and resistance is increasing worldwide [18–20]. For example, the *U. urealyticum* resistance rate to ofloxacin increased from 24.1% in 1999to 66.8% in 2004 [18]. *U. urealyticum* and *M. hominis* had resistance rates of 50.6% and 17.7% to ofloxacin, respectively, between 2011 and 2015 [20]. Consistently, in this study, ofloxacin, sparfloxacin, and levofloxacin, which are all quinolones, were the top three antibiotics associated with resistance, accounting for approximately 50% of the total resistance; their rates were much higher than those associated with tetracyclines and macrolides. Notably, there was a gradually increasing prevalence of quinolone resistance from 2015 to 2018. The mechanism of quinolone resistance involves mutations in the gyrA and gyrB subunits of DNA gyrase and parC and parE subunits of topoisomerase IV that probably arise because of antimicrobial selective pressure [17, 21]. Wang et al. reported that nemonoxacin, a novel nonfluorinated quinolone, may have increased antibacterial activity and decreased adverse effects on account of its C-8-methoxy structure on the quinolone ring [22]. The resistance rates for tetracyclines including tetracycline, doxycycline, and minocycline remained below 3%, and the resistance rates for macrolides including josamycin, roxithromycin, clarithromycin, and azithromycin were approximately 20%. Acquired tetracycline resistance is associated with the presence of the tet gene [23], while acquired macrolide resistance is related to mutations in the 23S rRNA region and variations in ribosomal proteins L4 and L22 [24].

The general increasing trend of antibiotic resistance over the past four years is concerning, and none of the antibiotics showed an obvious decreasing trend. A position statement from the European Sexually Transmitted Infection Guidelines Editorial Board noted that routine detection and treatment of asymptomatic or symptomatic genital *Mycoplasma* infection in men and women are not recommended because the majority of individuals with asymptomatic infection by these bacteria do not develop disease and excessive detection and treatment may result in the development of antimicrobial resistance [25]. However, a few studies have investigated unresolved issues regarding genital *Mycoplasma* infection and its independent relationships with sexual risk behaviors, which can result in sexually transmitted diseases (STDs), and/or prostitution [26] and complications such as infertility and pelvic inflammatory disease [27, 28]. Antibiotic treatment can eliminate genital *Mycoplasma* infection; however, elimination failure may lead to persistent symptoms and signs. Therefore, high-risk populations, such as sex workers and symptomatic patients with genital *Mycoplasma* infection, should be treated with appropriate antibiotics after informed consent is obtained.

## 5. Conclusions

In conclusion, this study showed increasing trends in the prevalence of infection and levels of antibiotic resistance in *U. urealyticum* and *M. hominis* from 2015 to 2018, which clearly confirms the necessity to monitor the standardized administration of antibiotics.

## Figures and Tables

**Table 1:** Clinical characteristics of gynecological outpatients.

Characteristics	Total	*Mycoplasma*		OR	95% CI	*P*
		Positive (*n* = 3205)	Negative (*n* = 2465)			
*Age*						
≤19	57	26 (0.8)	31 (1.3)	Reference		0.019
20–29	2831	1608 (50.2)	1223 (49.5)	1.568	0.926–2.654	
30–39	1660	947 (29.5)	713 (28.9)	1.584	0.932–2.691	
40–49	1032	587 (18.3)	445 (18.1)	1.573	0.921–2.687	
≥50	90	37 (1.2)	53 (2.2)	0.832	0.426–1.626	

*Marital status*						
Married	4652	2561 (79.9)	1991 (80.8)	Reference		0.417
Unmarried	1018	644 (20.1)	474 (19.2)	1.056	0.925–1.206	

*Education*						
High school and below	2374	1632 (50.9)	1142 (46.3)	Reference		0.003
Bachelor	2239	1216 (38.0)	1023 (41.5)	0.832	0.743–0.931	
Master and above	1057	357 (11.1)	300 (12.2)	0.833	0.702–0.988	

*Occupation*						
Yes	4371	2468 (77.0)	1903 (77.2)	Reference		0.862
No	1299	737 (23.0)	562 (22.8)	1.011	0.892–1.146	

*Smoking*						
No	86	3048 (95.1)	2336 (94.8)	Reference		0.568
Yes	5584	157 (4.9)	129 (5.2)	0.933	0.734–1.185	

*Drinking*						
No	632	2631 (82.1)	2007 (81.4)	Reference		0.517
Yes	5038	574 (17.9)	458 (18.6)	0.956	0.835–1.095	

*Gravidity*						
0	1040	781 (24.4)	659 (26.7)	Reference		0.043
≥1	4630	2424 (75.6)	1806 (73.3)	1.133	1.004–1.277	

*Parity*						
0	2156	1195 (37.3)	961 (39.0)	Reference		0.191
≥1	3514	2010 (62.7)	1504 (61.0)	1.075	0.965–1.197	

*Menopause status*						
No	297	3055 (95.3)	2318 (94.0)	Reference		0.032
Yes	5373	150 (4.7)	147 (6.0)	0.774	0.613–0.978	

*History of gynecological diseases*						
No	2361	1813 (56.5)	1496 (60.7)	Reference		0.002
Yes	3309	1392 (43.5)	969 (39.3)	1.185	1.065–1.319	

**Table 2:** Multivariate logistic regression analysis of risk factors for *Mycoplasma* isolation.

	*β*	SE	Wald	Sig.	OR	95% CI
*Age*						
≤19	Reference					
20–29	0.438	0.270	2.625	0.105	1.549	(0.912, 2.631)
30–39	0.407	0.273	2.219	0.136	1.502	(0.879, 2.565)
40–49	0.394	0.278	2.002	0.157	1.483	(0.859, 2.559)
≥50	−0.193	0.376	0.264	0.608	0.825	(0.395, 1.722)

*Education*						
High school and below	Reference					
Bachelor	−0.196	0.058	11.596	0.001	0.822	(0.734, 0.920)
Master and above	−0.217	0.088	6.085	0.014	0.805	(0.678, 0.956)

*Gravidity*						
0	Reference					
≥1	0.102	0.064	2.549	0.110	1.107	(0.977, 1.255)

*Menopause status*						
No	Reference					
Yes	−0.157	0.154	1.037	0.309	0.854	(0.631, 1.157)

*History of gynecological diseases*						
No	Reference					
Yes	0.182	0.057	10.230	0.001	1.200	(1.073, 1.341)

**Table 3:** Yearly distributions of *U. urealyticum* and *M. hominis* from 2015 to 2018.

Year	Total	*U. urealyticum*	*M. hominis*
2015–2018	5670	3205 (56.53)	625 (11.02)
2015	1251	673 (53.80)	109 (8.71)
2016	1275	704 (55.22)	128 (10.04)
2017	1509	858 (56.86)	185 (12.26)
2018	1635	970 (59.33)	203 (12.42)
*χ* ^*2*^	—	20.16	27.67
*P*	—	0.018	0.003

**Table 4:** Antibiotic resistance of *U. urealyticum* and *M. hominis* from 2015 to 2018.

Antibiotic	2015–2018	2015	2016	2017	2018	*χ* ^*2*^	*P*
Tetracycline	77 (2.40)	12 (1.78)	14 (1.99)	24 (2.80)	27 (2.78)	2.786	0.426
Ofloxacin	1982 (61.84)	247 (36.70)	388 (55.11)	593 (69.11)	754 (77.73)	316.8	<0.001
Doxycycline	51 (1.59)	12 (1.78)	7 (0.99)	13 (1.52)	19 (1.96)	2.628	0.453
Josamycin	32 (1.00)	6 (0.89)	4 (0.57)	10 (1.17)	12 (1.24)	2.198	0.532
Sparfloxacin	1652 (51.54)	228 (33.88)	364 (51.70)	514 (59.91)	545 (56.19)	116.5	<0.001
Roxithromycin	510 (15.91)	57 (8.47)	84 (11.93)	172 (20.05)	197 (20.31)	61.17	<0.001
Minocycline	48 (1.50)	9 (1.34)	9 (1.28)	15 (1.75)	15 (1.55)	0.728	0.867
Levofloxacin	1298 (40.50)	108 (16.05)	189 (26.85)	420 (48.95)	581 (59.90)	398.3	<0.001
Clarithromycin	401 (12.51)	48 (7.13)	69 (9.80)	144 (16.78)	140 (14.43)	40.09	<0.001
Azithromycin	454 (14.17)	49 (7.28)	75 (10.65)	160 (18.65)	169 (17.42)	56.12	<0.001

## Data Availability

The datasets used or analyzed during the current study are available from the corresponding author on reasonable request.
